# High-throughput Evaluation of Protein Migration and Localization after Laser Micro-Irradiation

**DOI:** 10.1038/s41598-019-39760-8

**Published:** 2019-02-28

**Authors:** Sebastian Oeck, Nathalie M. Malewicz, Adam Krysztofiak, Audrey Turchick, Verena Jendrossek, Peter M. Glazer

**Affiliations:** 1 Department of Therapeutic Radiology, Yale University School of Medicine, New Haven, CT 06520 USA; 2 Institute of Cell Biology (Cancer Research), University Hospital Essen, University of Duisburg-Essen, Essen, 45122 Germany; 3 Department of Anaesthesiology, Yale University School of Medicine, New Haven, CT 06520 USA; 4 Department of Genetics, Yale University School of Medicine, New Haven, CT 06520 USA

## Abstract

DNA- and histone-related research frequently comprises the quantitative analysis of protein modifications, such as histone phosphorylation. Analysis of accumulation and disappearance of protein foci are used to monitor DNA damage and repair kinetics. If the protein of interest doesn’t accumulate in foci, laser micro-irradiation of single nuclei provides an alternative method to monitor DNA repair proteins and histone dynamics at the DNA damage site. We have developed an automated evaluation tool for standardized, high-throughput analysis of micro-irradiated cells featuring single cell background subtraction and detection across multiple fluorescence channels, allowing for robust statistics.

## Introduction

When characterizing the cellular DNA damage response to environmental compounds, chemotherapy, radiation, or other DNA damaging agents, nuclear protein localization is an effective and widely used method to analyse DNA repair kinetics by detecting changes in chromatin structure and protein localization. One procedure is the analysis of nuclear foci based on fluorescence microscopy of fusion proteins, direct fluorescence staining, or an immune approach^1–4^. For example, the detection of phosphorylated histones, like H2A.X, and the monitoring of accumulation and disappearance of DNA repair protein foci is used to analyse DNA damage and repair kinetics^3,5^. However, the analysis of foci formation or other localization-based maxima requires that the protein of interest forms these foci; otherwise, a different methodological approach is needed^4,6–8^.

Laser micro-irradiation was first mentioned in scientific literature in the late sixties by Amy, Storb, Wertz and co-workers^9^. Furthermore, Berns *et al*. refined the method and used it for a variety of setups including single cell and chromosome irradiation^10,11^. With the development of fluorescence-based microscopy, the technique evolved to a modern method commonly used in the field of radiation and DNA repair research as well as cell and cancer biology. The laser micro-irradiation of cells is an increasingly used method in the field with more and more ways of application (243 articles in total and 123 articles in combination with the term DNA repair, with 46% published between 2014 and 2018). Laser micro-irradiation allows analysing the accumulation of the protein of interest or its modification (e.g. phosphorylation, methylation, acetylation), even if these events don’t manifest as foci and just result in an increased presence of the protein or protein modification at the DNA damage region^4,7,8,12^. However, the analysis of large sets of images and the resulting datasets from cells with defined micro-irradiated areas is still challenging, time-consuming and often performed as qualitative analysis, not statistically meaningful, as there is no tool available for the analysis. Following the current practice, usually a single or a handful of cells is analysed to determine the fluorescent intensity in a restricted region of the cell^8,13^. Some approaches have been developed to analyse the intensity or area under the curve of micro-irradiated regions, although these are performed mainly manually in a qualitative manner^14–16^ or require live cell monitoring of the irradiated sample mounted on a fluorescence microscope^15,17^.

## Results and Discussion

**Figure 1:**
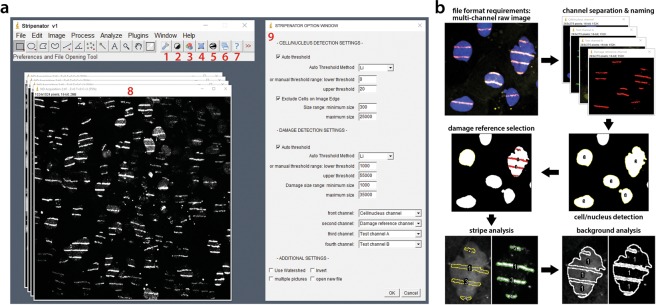
Interface and evaluation procedure. (**a**) The interface embedded in the ImageJ software with seven shortcut buttons for separate procedures of the Stripenator (1–7), an example stripe image (8) and Stripenator option window (9). The button shortcuts consist of the options (1), four buttons for testing the preferences in an example picture, such as a nucleus thresholding button (2), watershedding for nuclei (3), nucleus selection (4), thresholding of stripes (5), additionally an analyse button to run the whole analysis procedure in one or multiple images (6) and a help button (7). The *Stripenator Option Window* (9) offers adjustment for selection of nuclei and stripes. (**b**) The Stripenator workflow allows the user to analyse damaged areas and their corresponding background values by marking the damage and non-damage areas. The images show DAPI-stained nuclei in blue and γH2A.X DNA damage reference in red.

We have developed an ImageJ-based, user-friendly, high-throughput evaluation tool, the Stripenator, to standardize and accelerate the quantitative analysis of local protein accumulation at sites of DNA damage. One of the major strengths of the tool is the statistically valuable threshold of analysed cells is achieved through an objective evaluation process. The installation of the tool is simple, and the interface is self-explaining (Fig. 1a). Importantly, results obtained by a novice user and an expert are comparable.

Our tool uses the *Thresholding* and *Analyze Particles* ImageJ procedures, enabling an automated detection of nuclei and stripes or other regions of interest (ROI) (Fig. 1b). In the second step, it gathers information of these ROIs such as shape, size and grey scale data (mean intensity, etc.) using the *Measure* command in up to two channels and the damage reference channel e.g. γH2A.X or phospho-53BP1 fluorescence channel. Evaluating the data of non-damaged and damaged areas, it is possible to get distinct data of single cell damage area and its own background in each channel. These ROIs can be selected with a pre-set size and intensity thresholding values using different automated thresholding methods or by a manual threshold range. For a separation of overlapping cells, the *Watershedding* procedure can be additionally used with the Stripenator. Selecting *multiple images*, the Stripenator can evaluate all images in one folder automatically without the presence of the user. Finally, the measured data is exported to an XLS spreadsheet (Microsoft Excel) for further analysis and the analysis parameters for each image documented in another file.

The import into a Microsoft Excel spreadsheet enables further sorting, statistical analysis and easy export into other statistics software. Each file includes area measured in pixel count, mean intensity named “mean”, standard deviation “StdDev” of the intensity, maximum, minimum intensity. These values are measured in each channel separately for each cell/nucleus and each test area per nucleus. Additionally, these values of the tested areas as well as the background areas are summed up and labelled by the cell number from each image. A detailed step-by-step manual including example image analysis and troubleshooting is provided in the supplement.

**Figure 2:**
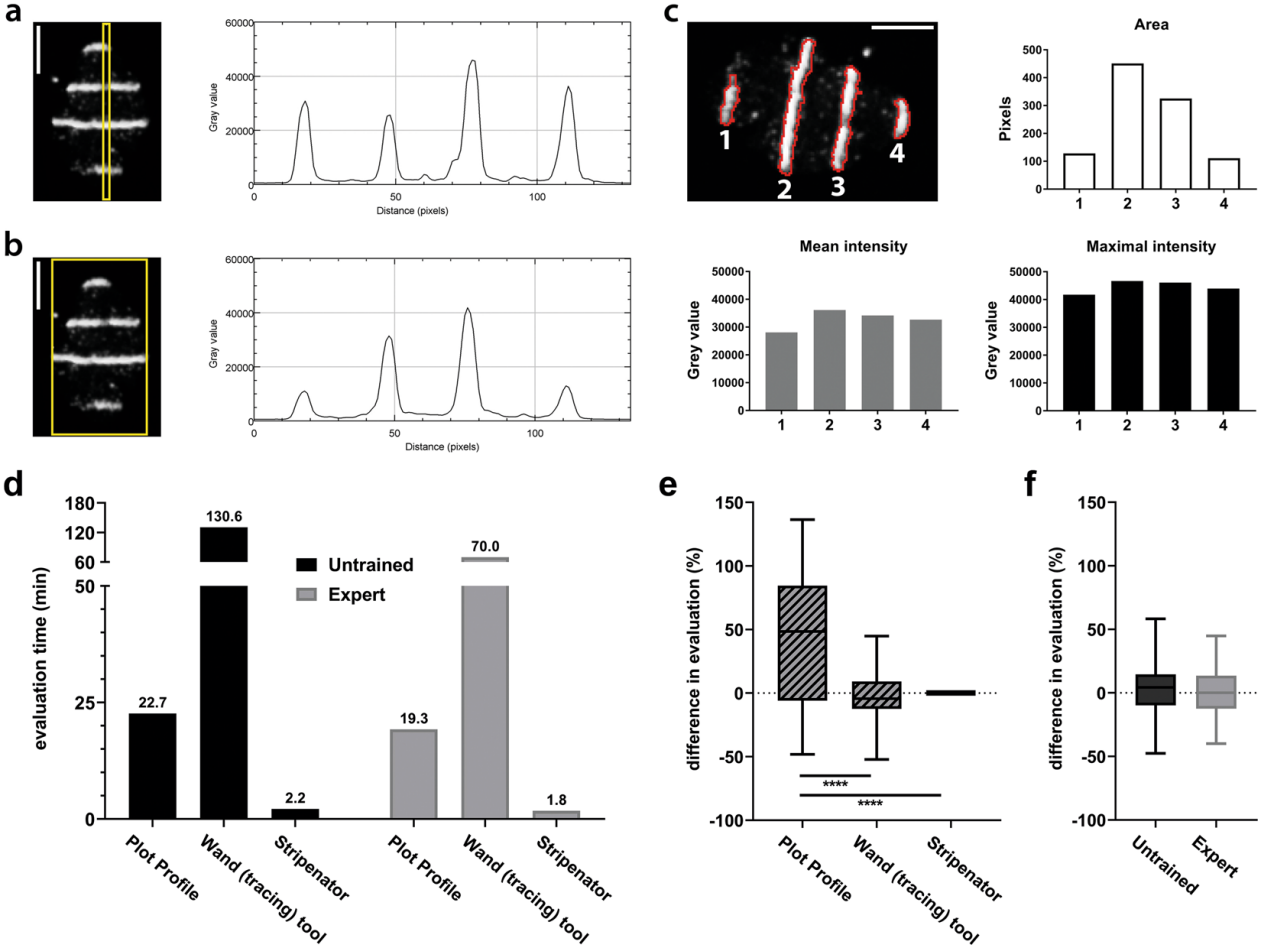
Reliability of values compared to manual ImageJ-based evaluation. (**a**,**b**) The manual analysis of a narrow area over a single nucleus using the ImageJ *Plot Profile* function varies considerably from the plot generated from a whole nucleus frame. The scale bar represents 5 µm. (**c**) The Stripenator analyses a variety of values of each damage area (1–4) including area size, mean and maximal intensity. These values are measured independently of the size, angle and position of the damage areas. The images show the area of a single nucleus with a γH2A.X DNA damage reference staining. The scale bar represents 5 µm. (**d**) Analysis of evaluation time was performed by two users with three different methods. User-variability was measured as difference of values between two users using the three different analysing methods (**e**) and between the method *wand (tracing) tool* and the Stripenator for an untrained user and an expert (**f**).

A commonly used method for qualitative analysis of irradiated regions is the ImageJ function Analyze/Plot Profile. This function generates a plot of intensity against pixel distance (Fig. 2b). These plots can also be used for quantitative analysis by maximal intensity or area under the curve measurements, such as in the literature commonly used analysis of western blot signals. Usually, it is necessary that the marked area is wider than one pixel to receive a more realistic mean intensity (Fig. 2b). ImageJ will then average the intensity value over the whole width of the ROI (yellow rectangle). In our example, the values of Fig. 2a,b for the third peak match almost perfectly but plotting the whole area changed the values of the other three peaks since the yellow frame is larger than the nucleus. Because the Plot Profile function requires a rectangular shaped area, it is not possible to use a nucleus shaped ROI. Furthermore, cell movement might turn the areas out of line and bias the results. Another source of bias can be introduced by the investigator subjectively choosing a wider or less wide analysis area. Especially for the first method, which uses marking of only a small rectangular sector inside the nucleus choosing areas with intensity can cause differing results, as shown in the example (Fig. 2a,b).

Other groups have tried to avoid this issue by using stripe micro-irradiation and a rotation of the single cell image to compensate for possible cell movement. Another approach is the manual selection of nuclei and damage areas in thresholded images with ImageJ’s *wand (tracing) tool*. The Stripenator is unaffected by shape or angle of the area of interest and automatically selects these areas. Each tested damage area of a cell/nucleus is analysed independently and additionally values for combined damage areas are measured (Fig. 2c). Depending on the needs, the produced output spreadsheet gives values which can be sorted and analysed in different ways. One approach for a reasonable evaluation of micro-irradiated regions is to use the mean intensities and StdDev and compare these to the related background areas, as can be done manually with the *wand (tracing) tool* of ImageJ or the Stripenator. The fold-change between tested areas and corresponding background reveals the change of the tested signal like migration or activation of DNA repair proteins to/in the damage site. Thereby, different timepoints after damage induction can describe a kinetic of the tested event. In addition to the intensity data, the Stripenator reports size of micro-irradiated areas and their shape. This feature represents an important advantage over conventional manual analysis. Furthermore, analysis of micro-irradiation can be subjected to investigator-related bias, since only a small number of cells is analysed and might be selected manually. The Stripenator can analyse hundreds of cells in minutes. We analysed and compared the evaluation speed and user variability for different methods performed by an untrained user and an expert. Using the Stripenator, both were able to analyse the same image in a fraction of the time needed when using commonly used rectangle average method or the *wand (tracing) tool* (Fig. 2d)^7,8,12,13,15^. The Stripenator also offers a higher accuracy than the *Plot Profile* method and achieves results comparable to the *wand (tracing) tool*. The Stripenator’s results were independent of the training status of the user (Fig. 2e,f). Consequently, a more meaningful analysis and statistical evaluation can be performed with the acquired images.

**Figure 3:**
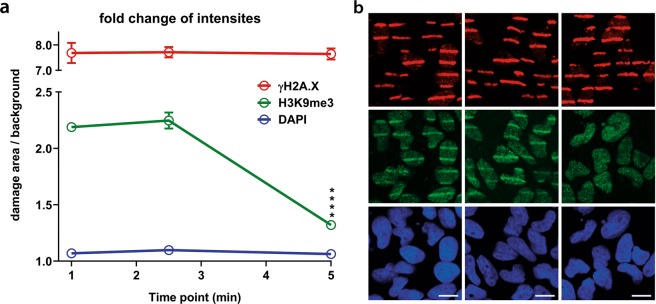
H3K9me3 demethylation example kinetic. (**a**) Evaluation of H3K9me3 and γH2A.X in HeLa cells over time. Data consist of at least 50 cells per replicate, n = 3 ****p < 0.001. (**b**) Example images of γH2A.X (red), H3K9me3 (green) and DNA (blue) at 1, 2.5 and 5 min after laser micro-irradiation. The scale bar represents 10 µm.

To further validate and demonstrate the applicability of the Stripenator, we performed an example analysis of the demethylation of the trimethyl histone 3 lysine 9 (H3K9me3) in HeLa cells over time (Fig. 3). The damage reference γH2A.X was measured on a constant high level, whereas the areas of the damage reference showed no difference in intensity compared to the background in the DAPI channel. Furthermore, we were able to analyse the rapid demethylation of H3K9me3 between 1 and 5 min after DNA damage induction by laser micro-irradiation.

The Stripenator is the first automated tool developed to analyse defined areas of laser micro-irradiation on a single cell level. It represents a user-friendly tool for fast high-throughput analysis and data export to Microsoft Excel. In comparison to manual analysis, it overcomes investigator-related bias and extremely reduces analysis time. Furthermore, it provides additional options for damage area analysis like size and intensity of nuclear and damage area as well as the corresponding background areas. The background subtraction represents a significant advantage of this method, leading to realistic data representation of the actual signal above background and a consistency across data sets and experiments. Moreover, data evaluation becomes easier to reproduce and less biased, analysis preferences can be documented, and evaluation can be repeated by different experimenters leading to the same results. A novel achievement is that the analysis can be performed on a statistically meaningful number of cells, not only handpicked example cells, in a brief time allowing for scientific rigor and quantitative statistical analysis.

Laser micro-irradiation is a widely used approach having led to meaningful findings in cell biology, aging and cancer research^7,8,18,19^. With all its advantages over current existing manual evaluation tools, the Stripenator provides value and utility in objective, fast analyses and enables superior quality, high-throughput studies of migration and accumulation of DNA damage response factors, histone modifications, and DNA repair-related proteins. These capabilities will facilitate a deeper understanding of a broad range of cellular mechanisms, from fundamental studies to support of preclinical research for translation into clinical application.

## Methods

### Literature search strategy

A PubMed search was conducted to assess the significance and development of publications in the field of micro-irradiation. A search for the terms- “laser microirradiation OR laser micro-irradiation OR microirradiation OR micro-irradiation” and for the search terms “(laser microirradiation OR laser micro-irradiation OR microirradiation OR micro-irradiation) AND (DNA repair OR DNA damage OR double-strand break)”. Sources published until 12/31/2018 were included. The first published articles were registered 1959.

### Cell culture and BrdU sensitization

Sterile grid cover slips (10816; ibidi inc.) were placed in 50 mm glass-bottom dishes (P50GC-1.5-11.5–14-F; MatTek corp.) and coated overnight with 20 µg/mL Collagen A (A1048301; Thermo Fisher Scientific) in PBS at 4 °C. Subsequently, exponentially growing HeLa cells (CCL-2; ATCC) were plated onto the cover slip (300,000 to 400,000 cells per dish) in DMEM medium (11965; Thermo Fisher Scientific) supplemented with 10% FBS at 37 °C in a humified 5% CO_2_ atmosphere. After one day 10 µM BrdU (19–160; EMD Millipore) was added to the culturing medium and cells were incubated additional 48 h prior to irradiation.

### Laser micro-irradiation

Shortly before the irradiation, cells were washed with PBS and the medium was replaced with warm low absorption medium (31053; Thermo Fisher Scientific). Cells were placed in an incubator chamber at 37 °C with 5% CO_2_ supplementation mounted on a Leica TCS SP8 X microscope system (Leica Microsystems). A Leica HC PL APO 40 ×/1.30 Oil CS2 objective and a zoom of 0.75 made it possible to irradiate up to 200 cells in one frame based on the cell size and the seeding. We placed 40 horizontal stripe masks (5 px wide) in a view field of 512 × 512 px and irradiated the cells 35 times with a 405 nm diode laser at 95% with FRAP booster with a pixel dwell time of 3.75 µs. One full frame irradiation lasted 1.985 s. Thus, a total irradiation with 35 iterations took 69.3 s (130 Hz bidirectional, frame rate 0.503/sec). We measured a laser power of 1.14 J/sec exiting the objective. Consequently, each pixel of the irradiation masks was exposed to 4.275 µJ per iteration resulting in a total energy of 149.625 µJ. After irradiation, cells were fixed and permeabilized with 3% formaldehyde, 0.2% Triton X-100 and 8% sucrose for 15 min at RT.

### Immunofluorescence staining

We used following primary and secondary antibodies: anti-γH2A.X (9718 S; Cell Signaling; 1:400), anti-trimethyl H3K9 (05–1250; EMD Millipore; 1:200), anti-rabbit Alexa Fluor Plus 488, anti-rabbit Alexa Fluor Plus 555 and anti-mouse Alexa Fluor Plus 555 (A32731, A32732, A32727; Thermo Fisher Scientific; 1:400). After overnight blocking in 5% normal goat serum with 0.2% Triton X-100 in PBS at 4 °C, cells were incubated with primary antibodies in blocking buffer for 2 h at RT. Following 3 washing steps with PBS + 0.5% Triton X-100, cells were stained with secondary antibodies in blocking buffer for 2 h. DNA was stained with DAPI (1816957; Thermo Scientific Inc ; 2.5 µg/mL) for 20 min at RT. Samples were washed twice with PBS + 0.5% Triton X-100 and then rinsed one time with PBS before mounting with DAKO Fluorescence Mounting Medium (S3023; Dako NA Inc.). Images were analysed with a Nikon Eclipse Ti fluorescence microscope with a Plan Fluor 40 × /1.30 Oil DIC N2 objective, a CSU-W1 confocal spinning disk unit, an iXon Ultra camera (Andor Technology), MLC 400B laser unit (Agilent Technologies) and NIS Elements 4.30 software (Nikon Corporation).

### Software and programming

The “Stripenator”, an ImageJ macro-based software solution was developed based on the reliable structure of the Focinator, a tool for automated high-throughput analysis of foci^1,2^. ImageJ is an open-source software for image processing and analysis providing possibilities to automate and extend procedures via macros and plugins (the National Institutes of Health, Version 1.5) (NIH)^20^. The plugin used by the Stripenator is the Bio-Formats importer plugin enabling import of multichannel files (version 5.8.2)^21^.

### Installation procedure

For the analysis with the Stripenator, ImageJ is needed. The computer requirements are comparable with these of ImageJ. A faster computer set-up might result in an increased evaluation speed. The speed and variability tests (Fig. 2d,e) were performed by an untrained user and an expert on the same system (Dell XPS 15 with Intel Core i7-7700HQ @ 2.80 GHz, 32 GB RAM, Toshiba XG3 1TB M.2 NVMe PCIe SSD, Intel HD Graphics 630, Windows 10 Home 64-bit v1803). Multi-channel images, such as ZVI, CZI, LIF or TIFF files, are necessary. The Stripenator also requires the ImageJ Bio-Formats plugin to open these file formats. The Stripenator macro, instructions and support are obtainable at https://www.focinator.com/stripe and in the supplement. The Stripenator macro can be installed as a Startup Macro (“Plugins/Macros/Startup Macros….”) by copying the whole text of the Stripenator.txt file into the text editor window of the Startup Macro window. After saving the new macro, ImageJ needs to be relaunched and the analysis can then be started.

### Structure and application

The tool employs mainly *Thresholding* in different channels to contrast structures for detection and *Analyze Particles* for automated detection of ROIs based on size and intensity. The selected areas are saved as a mask and then the analysis using the *Measure* command is performed on the original, unchanged images in each channel exporting shape, size and grey scale data, such as mean intensity, in the damaged area, the undamaged area as background and over the whole nucleus. The Stripenator’s interface consists of seven shortcut buttons and a menu (Fig. 1a). For adjusting the settings of analysis and selecting a new file press <F1> *Preferences and File Opening* (button 1) to open *Stripenator Option Window*. This window gives the user various options for setting the *Cell/Nucleus Detection* including thresholding settings, either by pre-set automated methods, like *Li* and *Huang*, or by setting a threshold range as well as a range for the tolerated cell or nucleus size. Furthermore, it is possible to adjust the *Damage Reference Detection* threshold and damage area size for the damage reference channel, e.g. γH2A.X-stained stripe areas. The order of the channels needs to be selected to define the *Cell/nucleus channel*, the *Damage reference channel* and the two *Test channels*. But it is also possible to use just one test channel. The downstream analysis is based on the order of the channels in the multi-channel image file. Further options can be selected, such as watershedding during the automated analysis, automatic analysis of multiple pictures, inverting in the case of images with inverted bit scale, and opening a new file using the Bio-Formats importer for the analysis or the first testing. The buttons 2–5 and shortcuts <F2 - F5> are used to pre-test the settings on an example picture.

The shortcut <F2> *Threshold Nuclei* (button 2) can be used for testing the correct threshold of the stained nuclei, usually the DNA channel, for the ROI detection. For most images with a good signal-background-ratio, we recommend first testing the images using the pre-set automated threshold methods *Li*, *Huang* or *Default*. In this procedure, the macro creates a duplicate of the channel and thresholds the image using the preferences set in the *Stripenator Options Window*. With <F3> *Watershed* (button 3) can be used to separate overlapping cells. With <F4> *Selecting Nuclei* (button 4), the thresholded ROIs are marked and then selected in the ROI manager for further analysis, for example *measure* commands to determine size and intensity of the nuclei. To detect damaged areas, the *Damage reference channel* needs to be selected by using <F5> *Threshold and Select Damage Area* (button 5). After the settings are adjusted, a new image can be opened using the *Stripenator Options Window* and either one or multiple images in one folder can be analysed with <F6> *Run All* (button 6). In the first step of the automated analysis, the software detects and selects the cells or nuclei (e.g. the DAPI staining for DNA/nucleus detection) in the pre-set ROI channel based on size and threshold setting. The next step marks the labelled micro-lesions in the damage reference channel, such as γH2A.X stained stripes or spots based on their pre-set size and threshold. To note, it is possible to analyse multiple damage areas in a single cell/nucleus. In the last step, the tool measures areas and intensities of the damage reference areas as well as the cells/nuclei and then subtracts stripes from the nucleus ROI to measure the background in all channels.

The macro can be run on a single file only, or in an automated mode which allows the user to assess a whole folder of image files. The automated mode should be used after having adjusted the parameters on at least one single picture. The results are saved in the same directory as “output” folder. The analysis of each image is saved in one file including the nucleus count and the measured values for each stripe, nucleus, and background in each channel. Additionally, for each image, the used preferences are saved in a text file and the ROI mask are saved in order to be able to comprehend the analysed nuclei.

### Evaluation time and variability

To evaluate the time needed to evaluate nuclei and damaged areas in 50 cells and the variability of the results with three different methods (single cell *Plot Profile* command of ImageJ resulting in an intensity histogram, the *wand (tracing) tool* method to acquire the intensities of a marked region and the Stripenator). Two users, independently and blinded to the experimental conditions performed the test evaluations (an untrained and in the field unexperienced person and an expert). Thresholding parameters for all measurements were pre-determined by the expert.

### Statistics

Kinetic experiments were analysed with two-way ANOVA with a Dunnett’s post hoc test. Variability between different user evaluations and different methods was calculated for 50 evaluated cells in one image as percentage of difference normalized to the average with a Bland-Altmann-Test. Statistical significance was determined with one-way ANOVA with Bonferroni post-hoc test. A priori alpha error p of less than 0.05 was considered statistically significant. Statistical analyses were performed using Prism8 (GraphPad Software) software.

### Online content

The Stripenator is freely available at our web page https://www.focinator.com/stripe.

## Supplementary information

Supplement
