# Supplementary material for: High-throughput Evaluation of Protein Migration and Localization after Laser Micro-Irradiation
Source: Sci Rep. 2019 Feb 28;9:3148. doi: 10.1038/s41598-019-39760-8 (PMC6395640; doi:10.1038/s41598-019-39760-8)
Supplement: Supplementary file 1 — Supplement [file 41598_2019_39760_MOESM1_ESM.docx]

**Supplementary Information**

**High-throughput Evaluation of Protein Migration and Localization after Laser Micro-Irradiation**

Sebastian Oeck^1,2,^*, Nathalie M. Malewicz^3^, Adam Krysztofiak^2^, Audrey Turchick^1^, Verena Jendrossek^2^ and Peter M. Glazer^1,4,^*

^1^ Department of Therapeutic Radiology, Yale University School of Medicine, New Haven, CT 06520, USA

^2^ Institute of Cell Biology (Cancer Research), University Hospital Essen, University of Duisburg-Essen, Essen, 45122, Germany

^3^ Department of Anaesthesiology, Yale University School of Medicine, New Haven, CT 06520, USA

^4^ Department of Genetics, Yale University School of Medicine, New Haven, CT 06520, USA

* To whom correspondence should be addressed: sebastian.oeck@yale.edu and peter.glazer@yale.edu

# **Stripenator | Step-by-Step manual v1.0**

## Introduction and general recommendations

For every analysis with the Stripenator please consider that the analysis can only be as good as the quality of your experiments and images. This is dependent on cell seeding, staining, microscope setup and finally image quality. The Stripenator works with many image file formats but needs multi-channel image files like ZVI, CZI, LIF, AF or TIFF. Raw data format should be used, because using jpgs or other compressed formats can distort the results. The raw format is based on your microscope camera. Usually these images possess more than 8-bit grey scale per channel (Fluorescence images are usually generated with a grey scale camera and have “no colour” in the raw format.) Furthermore, we recommend structuring the folders by for example cell lines or trial before producing the images and to only save images into the folders; consequently, the analysis will run more smoothly without further reorganization of your files.

In the following Step-by-Step manual, ImageJ *operations* and *windows* are formatted in *Italic*.

## Cell seeding

The seeding is based on many factors like cell proliferation rate, test dish/well size and surface, as well as experimental conditions including treatment and time points. Optimize the conditions to achieve a cell monolayer of about 70 to 80% cell confluency at the time of imaging. You might reduce the seeding if you plan longer time points after laser irradiation. Be aware of cells moving e.g. out of the irradiated frame. We would recommend point or square irradiation for long time points. Nevertheless, the Stripenator can still analyse cells with stripes which are turned in different directions in one image as a result of cell motility over time.

## **Installation and initialization of the Stripenator**

1. Install ImageJ bundled with Java 1.8.0 or higher. You can install different versions of ImageJ with different sets of Startup macros. (recommended folder: C:\ImageJ) (<https://imagej.nih.gov/ij/download.html>).
2. Install Bio-Formats Importer plugin (<https://www.openmicroscopy.org/bio-formats/>**)**
3. Open ImageJ and Open the *Startup macro window* by clicking on *Plugins/Macros/Startup Macros….*
4. Install the Stripenator macro by copying the whole text of the Stripenator.txt file into the open text editor window of the *Startup Macro window* in ImageJ. Click on *File/Save*.
5. Close ImageJ and relaunch. If the Stripenator was correctly installed a new set of buttons should appear as shown in Fig. 1a (1-7).

## **Adjusting the threshold and separation of cells/nuclei**

1. Click on the *wrench button* (Fig. 1a 1) to open the *Stripenator Option* w*indow* (Fig. 1a 9)
2. Adjust the parameters for thresholding, size and channel order to fit your cells and images. If you are unsure of these parameters, we suggest trying the pre-set parameters and check the box *open new file* and click *ok*. Please either check automated thresholding or uncheck and type in lower or upper thresholding values.
3. A new window will appear to select a file. Select a representative file for first parameter adjustment and testing.
4. The selected Image will be automatically opened with the Bio-Formats Importer. Remember the order in which the channels are opened by the Bio-Formats importer (Fig. 1a 8). The front channel is the channel on the top of the stack after opening the image with Bio-Formats. This is usually the channel with the highest number (Ch 3 or 4).
5. Select the DAPI or other nuclear staining channel (Supplement Fig. 1a 8) for testing and adjusting the threshold. For DAPI/Hoechst images with a normal signal/background ratio, the *Li* or *Huang* settings often do a good job. A good thresholding should keep the shapes of the cells/nucleus visible and distinguishable (Supplemental Fig. 1a 9), not produce holes in the cells (Supplemental Fig. 1a 10) or make the nuclei too large or artificially overlapping (Supplemental Fig. 1a 11).
6. Click on the *contrast button* (Supplemental Fig. 1a 2) to apply the preselected thresholding method and to create a duplicate with the potential thresholded mask (Supplemental Fig. 1a 9).

**Supplemental Figure 1.**


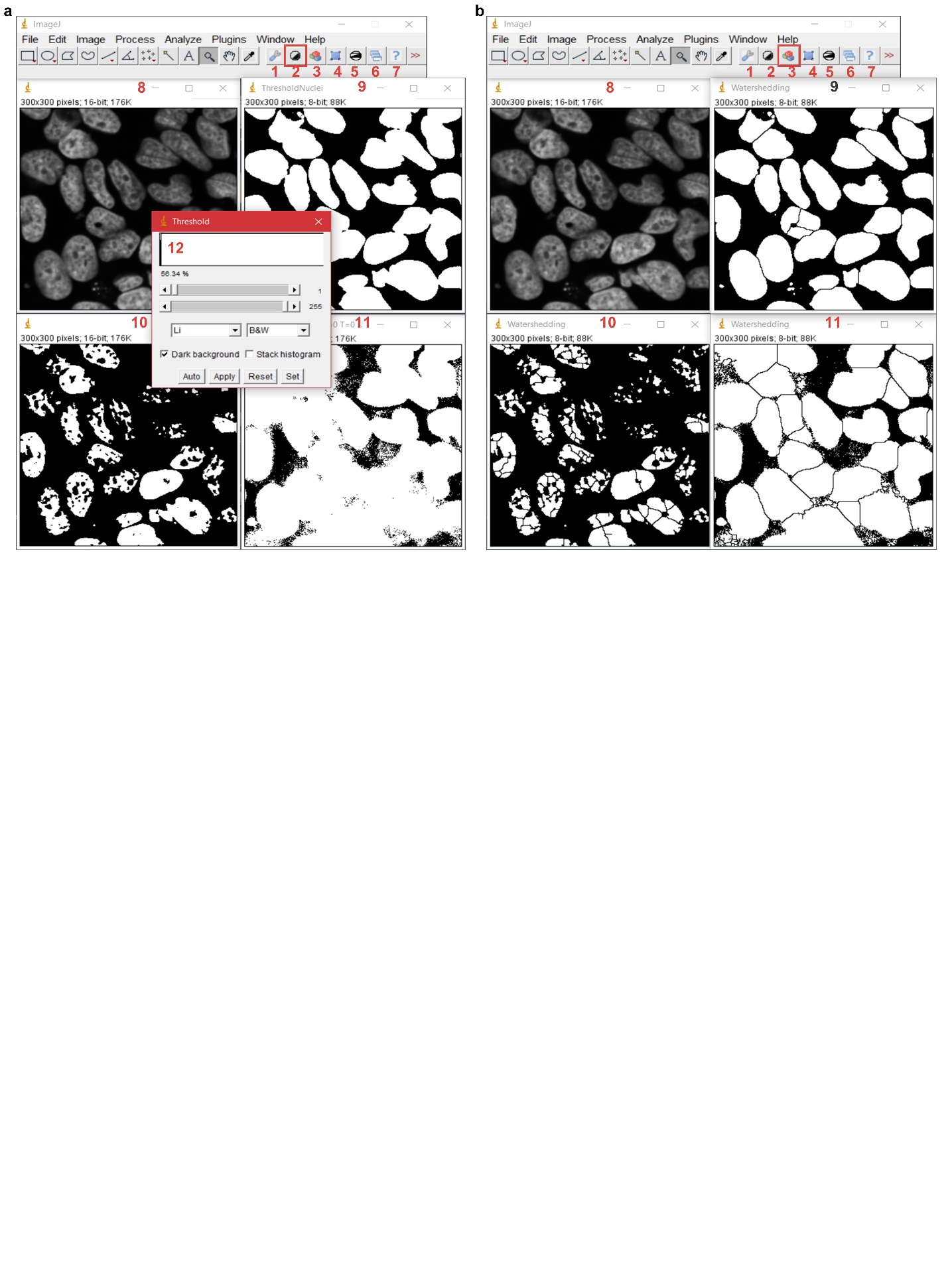


1. If the thresholding was not successful, repeat step 5-7 with adjusted parameters. You can also test the different threshold methods on a duplicated DAPI picture (*Image/Duplicate*). Use the *Threshold window* (*Image/Adjust/Threshold*) to test different auto threshold methods or manual values. Make sure *Dark background* is activated. Transfer your tested settings in the *Stripenator Option* *window* (Fig. 1a 9) and test again (step 6).
2. If nuclei are overlapping watershedding can be helpful. To test watershedding, select the image with the best thresholding result (Supplemental Fig. 1a 9) and click on the *brick stone button* (Supplemental Fig. 1b 3). A good example for watershedding is shown in Supplemental Fig. 1b 9. Supplemental Fig. 1b 10,11 show bad examples for watershedding based on a flawed thresholding.

## **Selecting cells/nuclei and damaged areas**

1. To test the size criteria of the cell/nucleus selection, click on either the thresholded image or the thresholded and watershedded image and click on the *blue selection rectangle button* (Supplemental Fig. 2a 4). A selection of the cells is being performed based on your size criteria in *Stripenator Option* *window* (Supplemental Fig. 3a 8). These regions of interest (ROIs) will be marked in your thresholded image (Supplemental Fig. 2a 9) and listed in the *ROI-Manager window* (Supplemental Fig. 2a 13). If incorrect size criteria are chosen, debris, partial cells or even background might be detected as ROI (Supplemental Fig. 2a 10) or smaller ROIs might be omitted (Supplemental Fig. 2a 11). If *exclude cell on image edge* was checked in the parameters, cells bordering the image will be ignored (Supplemental Fig. 2a 12).
2. You might determine the size of a big cell and a small cell to get an idea of the size using your thresholded image by using the *ROI-manager* and *Measure*. The *Result window* will show the size of the selected ROIs (Supplemental Fig. 2a 14). In case of the background being selected instead of the ROIs, please try the option *invert* in the *Stripenator Option window*.

**Supplemental Figure 2.**


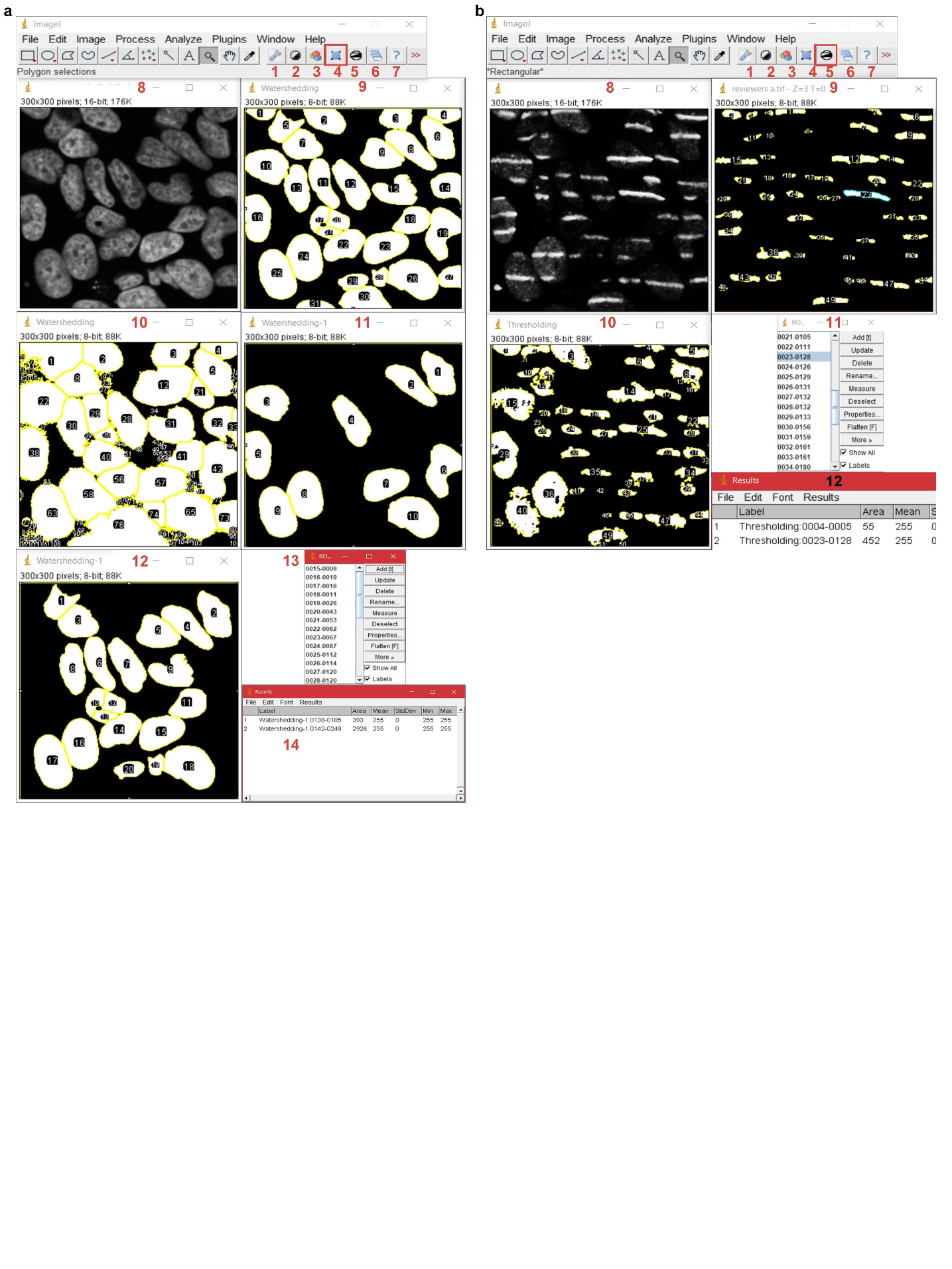


1. A similar approach can be applied for the detection of damaged areas e.g. stripes by choosing the threshold method and the size range of selected areas. The parameters for the damage detection settings can be also adjusted in the *Stripenator Option window (wrench button)*.
2. To apply and test the thresholding and selection parameters, choose the damage reference channel (Supplemental Fig. 2b 8) and click on the *contrasted rectangle button* (Supplemental Fig. 2b 5). A duplicate of the damage reference channel will appear based on the threshold and size settings (Supplemental Fig. 2b 9) and the damage reference ROIs are saved in the *ROI-Manager* (Supplemental Fig. 2b 11). If the damage area selection step was not fitting (Supplemental Fig. 2b 10), try other thresholding or size parameters similar done for the nuclei.

## **Running the automated analysis**

1. When all analysis parameters are determined close all images and channels. If using multiple picture analysis, the images should be similar regarding staining quality and microscope acquisition. To run the automated analysis, open the *Stripenator Option window* with the *wrench button* and apply the determined settings for the cell/nucleus and damage area detection (Supplemental Fig. 3a). Please remember to choose the correct order of the channels: One *Cell/nucleus channel* needs to be chosen, such as a DAPI staining, one *Damage reference channel*, such as γH2A.X, one analysis *Test channel A* and if a second channel for analysis is present this channel should be determined as *Test channel B*; if not present, the fourth channel must be *inactivated*.

**Supplemental Figure 3.**


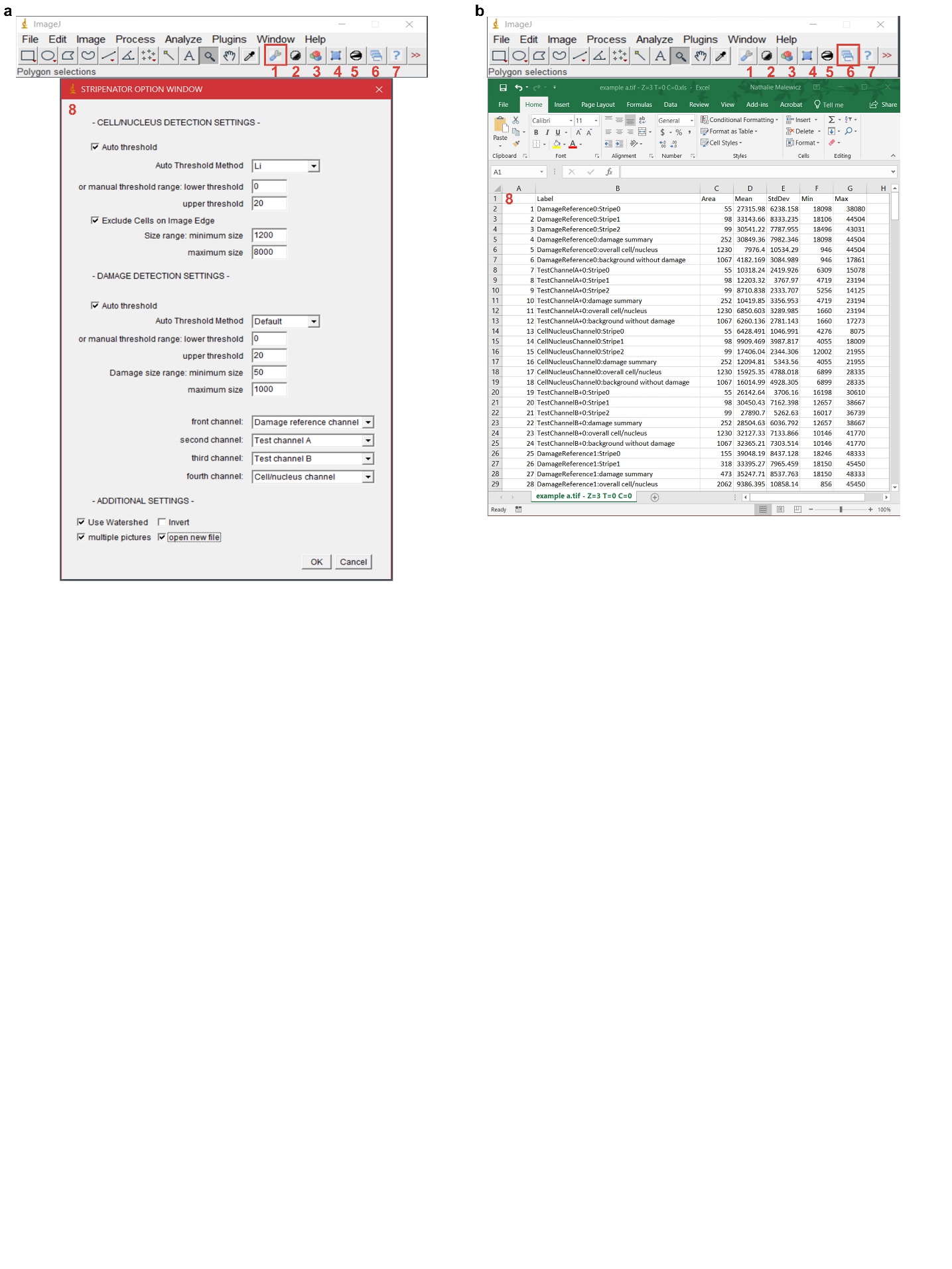


In the case of double channel selection or the channel number not fitting the actual channel number, an error message will appear after opening the image and starting the automated run. The user will have to correct accordingly. If only the order was chosen wrongly, no error will appear. Please pay attention to the correct order in which the Bio-Format importer opens the channels. If multiple images in one folder should be analysed with the same parameters, check *multiple pictures*. Check *open new file* and click *ok* to have a new window appear to select the image file or the first image file in a whole folder to be analysed. The example images can be tested with parameters shown in Supplemental Fig. 3a.

1. After the image is opened click on the *Run All* *button* (Supplement Fig. 3a 6) and observe the Stripenator performing the analysis by automatedly selecting channels, duplicating, thresholding, cropping, and closing images. Please wait until you are notified that the analysis is finished to obtain the results. If you detect wrong parameters, the analysis can be aborted with the “escape” key or by closing one of the windows.

## **Data extraction and interpretation**

1. After the automatic run, the result sheets for each image can be found in the output folder located in the same folder as the analysed images. There you will find a .zip file, a ROI mask that can be opened in the *ROI-Manager* to see which cells/nuclei were detected for each image, a .txt file with the chosen parameters and an .xls sheet with the output data.
2. To analyse the data, open the output .xls sheet and save as a new file with the .xlsx extension.
3. The output file shows the area, mean intensity, standard deviation of the mean intensity, min. and max. intensity per cell/nucleus, damage area and channel. For big files and easier handling, the labels can be copied, and files can be restructured with excel’s “find and replace” and “sorting and filtering” command or “text filters” (Supplement Fig. 3 b). Each row is named first with the channel name, then each damage area detected in a cell beginning with 0 or all damage area summarized in the “damage summary”, the “overall cell/nucleus” including damage area and background or only the “background without damage” per cell, followed by the cell number beginning with 0. If no damage was detected in the reference channel of the nucleus no damage summary and no damage area values are exported.
4. To obtain the mean intensities, for example for the *Test channel A,* results could be sorted for *Test Channel A* and damage summary and background without damage and the values copied. Another approach would be to calculate a damaged area/background intensity ratio for each cell to normalize the damage intensity values to their own background. Consequently, if the damaged and background area would have similar intensities because of no change, as for DAPI expected, a value of 1 would be obtained versus higher values if damage was more intensely stained than the background. Depending on the experimental setup other values such as the area or the intensity of each single damage area can also be analysed.
